# Plot-level estimates of aboveground biomass and soil organic carbon stocks from Nepal’s forest inventory

**DOI:** 10.1038/s41597-023-02314-9

**Published:** 2023-06-24

**Authors:** Shiva Khanal, Matthias M. Boer

**Affiliations:** 1 Forest Research and Training Center, Ministry of Forest and Environment, Kathmandu, Nepal; 2 Hawkesbury Institute for the Environment, Science Rd, Richmond, NSW 2753 Australia

**Keywords:** Forest ecology, Forestry

## Abstract

Given the contribution of deforestation and forest degradation to the global carbon cycle, forest resources are critical to mitigating the global climate change effects. Improved forest monitoring across different biomes is important to understand forest dynamics better and improve global projections of future atmospheric CO_2_ concentration. Better quantification of the forest carbon cycle advances scientific understanding and informs global negotiations about carbon emissions reduction. High-quality estimates of forest carbon stocks are currently scarce in many developing countries. Here, we present the most comprehensive georeferenced data set to date of plot-level forest carbon estimates for Nepal. Based on field observations from Nepal’s national forest inventory of 2010–2014; the data set includes estimates for two major forest carbon pools, aboveground biomass (AGB) and soil organic carbon (SOC) stocks from 2,009 and 1,156 inventory plots, respectively. The dataset fills an important knowledge gap about forest carbon stocks in the Central Himalayas, a region with highly heterogeneous environmental conditions and rich biodiversity that is poorly represented in existing global estimates of forest carbon.

## Background & Summary

The ‘Reducing Emissions from Deforestation and Forest Degradation’ (REDD+) initiative was proposed as a cost-effective climate change mitigation approach for developing countries to reduce carbon emissions and promote carbon sequestration through the financial incentive mechanism^1^. To comply with REDD+ reporting requirements and support national forest management, participating countries need accurate quantification of the reference level of national forest carbon stocks and monitor changes over time. Requirements for reporting changes in national carbon stocks, deforestation rates and emissions must be formalised and documented in a Monitoring, Reporting and Verification (MRV) system^2–4^. The MRV component of REDD+ deals with measurements of forest area change (activity data) and forest carbon stock changes (emission factors) that provide the basis for compiling a Greenhouse gas (GHG) inventory^5^.

Establishing an MRV system has been challenging for many developing countries, particularly in the tropics and mountainous regions, because of insufficient capacities to measure forests across highly heterogeneous national territories and monitor forest carbon stock changes continuously^6,7^. Besides institutional challenges for REDD+ programmes related to coordination, the role of stakeholders and regulatory frameworks^8^, the technical limitations in robust forest carbon monitoring constitute prominent challenges. With its mountainous topography, highly heterogeneous environment and diversity of forest types, Nepal exemplifies the significant challenges involved in producing periodic assessments of national forest resources.

**Fig. 1:**
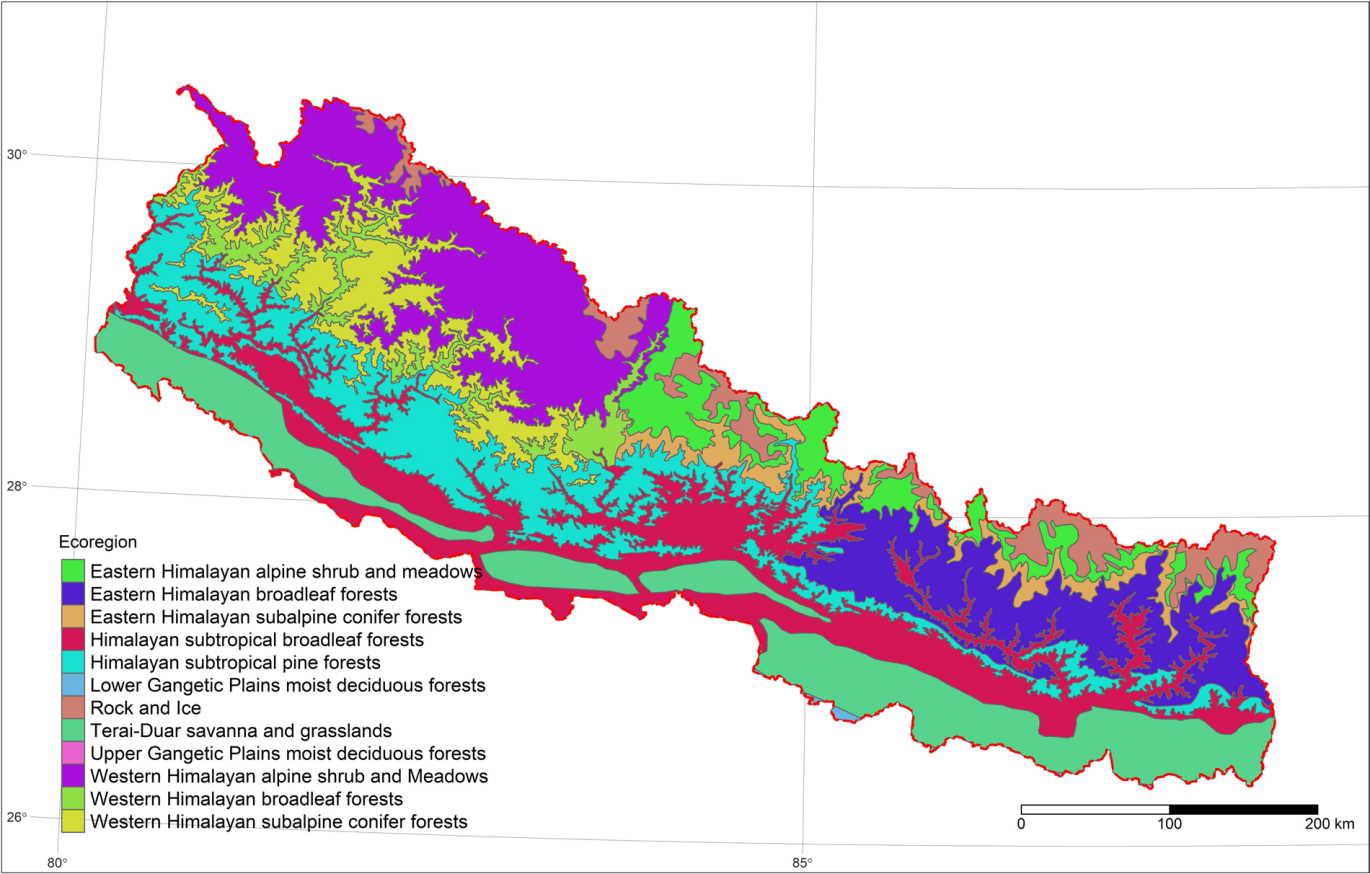
The map of terrestrial ecoregions in Nepal based on data from Olson^9^.

Nepal is located in the Central Himalayas, extending from 26° 20′ 53″ N to 30° 26′ 51″ N latitude and 80° 03′ 30″ E to 88° 12′ 05″ E longitude. The country covers a large elevational gradient from 59 masl in the southern Indo-Gangetic Plain to 8,848 masl at the top of Everest in the North. The pronounced topography and associated climate gradients/patterns sustain a highly diverse flora and fauna. According to the global ecoregion classification by Olson^9^, Nepal has 12 ecoregions, and a distinct feature of Nepal’s natural environment is the representation of a variety of those ecoregions within short distances (Fig. 1). The forested areas of Nepal cover a broad altitudinal gradient from less than 100 masl in the Gangetic Plain to the tree line of about 4,500 masl in the Himalayas^10^. Nepal has a forest area of 5.96 Mha, which is 40 percent of the total area of the country^11^. To accurately quantify and characterise spatial variation in forest carbon across this diverse region requires standardised observations across the full range of forest habitats.

The Intergovernmental Panel on Climate Change (IPCC) recognises five carbon pools: aboveground biomass, belowground biomass, litter, deadwood and soil organic carbon (SOC)^12^. Aboveground and belowground pools are the two main categories. The carbon held in the aboveground biomass (AGB) constitutes a significant portion of the terrestrial carbon pool^13^. When assessing forest carbon, AGB comprises all woody stems, branches, and leaves of trees, shrubs, and climbers. The belowground biomass carbon pools comprise fine and coarse tree roots and the microbial community and are difficult to quantify accurately, especially for forests with large trees or deep soils. Dead organic matter makes up the litter layers and coarse woody debris pool. The soil organic carbon (SOC) stock is another pool comprising organic carbon held in the soil below the litter^14^.

In this paper, we present the plot-level estimates of forest AGB and SOC for 1,127 plots, forest AGB only for 882 plots and SOC only for 29 plots. These estimates are thus available for a total of 2,038 sample plots, which were part of the permanent sample plots established for the national forest inventory. The field measurement of these inventory plots aimed for high consistency and accuracy and thus used collection protocols based on standard guidelines and trained and employed a large number of inventory crews comprising forestry graduates. This new data set provides the most comprehensive information to date on plot-level forest carbon estimates for all major physiographic regions of Nepal.

## Methods

**Fig. 2:**
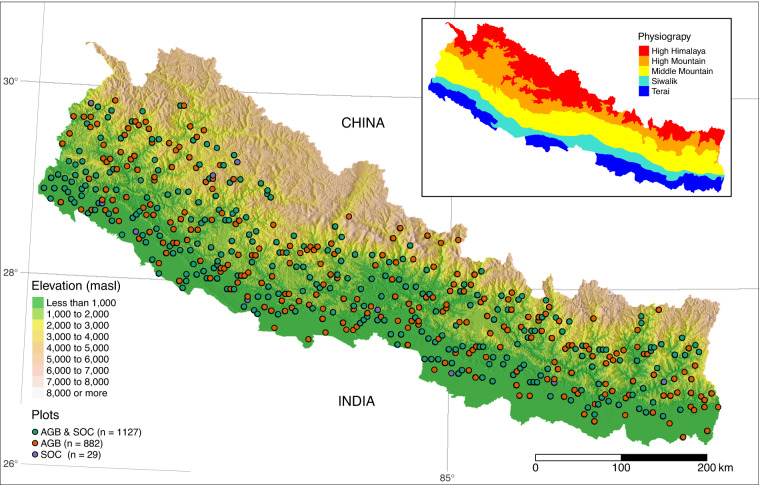
Elevation gradient, FRA plots and physiographic regions (inset) in Nepal.

The field observations used in this study were collected as part of the National Forest Resource Assessment (FRA) of Nepal by the Department of Forest Research and Survey (DFRS), Nepal, now restructured as the Forest Research and Training Centre (FRTC) between 2010 and 2014 (Fig. 2). The data collection followed standard protocols and was performed by trained professionals, including information on various aspects of the forest, such as trees, disturbance, soil, and other attributes. The details of the measurement protocols can be found in the standard field measurement protocol^15^. We used data related to trees, such as tree diameter and height, and soil sample analysis records, including bulk density, to estimate plot-level forest AGB and SOC. The main author served as one of the crew leader during the field measurement missions under National Forest Inventory to collect these datasets. This following subsections describe the sampling design, tree measurements, soil sampling, lab analysis protocols used by FRTC to prepare the raw data that we analysed to estimate plot-level forest AGB and SOC.

### Sampling design

**Fig. 3:**
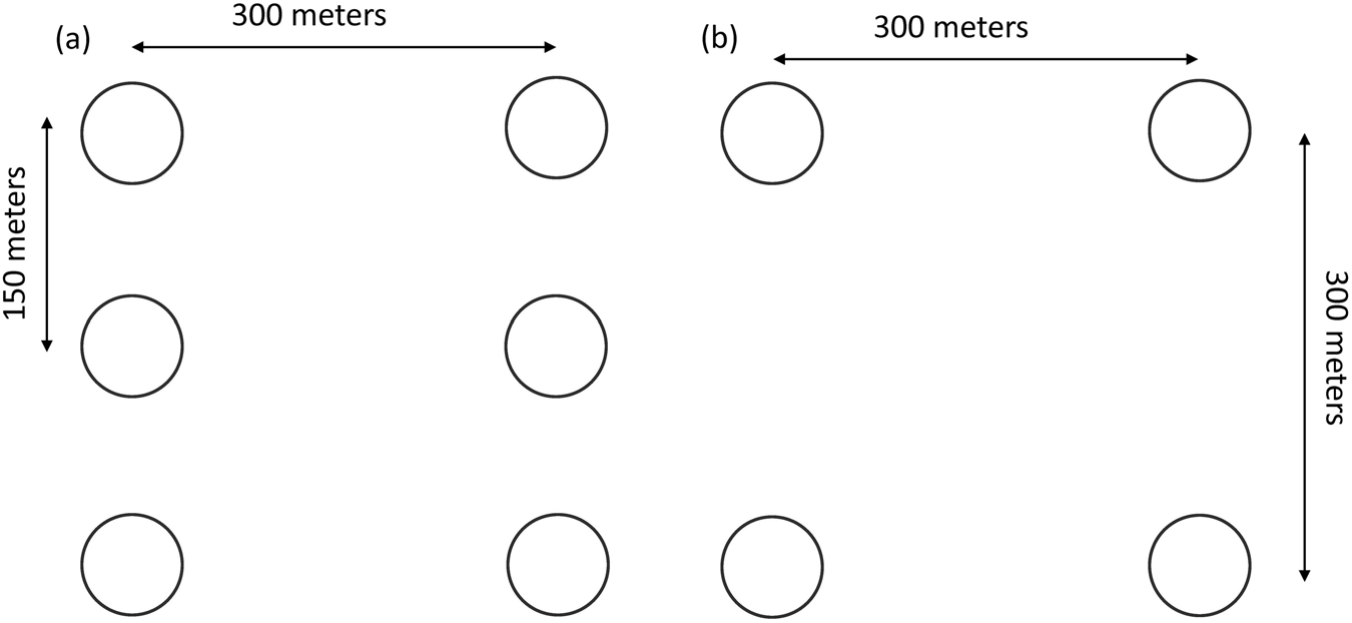
Cluster design used for the layout of sampling plots in (**a**) the four mountainous physiographic regions (Siwalik, Middle Mountain, High Mountain and High Himalaya) and (**b**) the Terai region.

The forest inventory design was based on the approach recommended by Kleinn^16^ and used in earlier national forest assessments of Nepal in the 1990s. The sampling approach was a two-phase stratified systematic cluster design. The first stratum consisted of five physiographic regions: Terai, Churia, Middle Mountains, High Mountains and High Himal (Fig. 2 Inset). Those regions are characterised by distinct geology, topography and climate, forming altitudinal belts that extend East-West, roughly aligned with the Central Himalayan mountain ranges. The first phase sample consisted of 9,230 clusters (55,358 plots total) laid out systematically on a 4 km square grid across the country. The land-use class of each plot in the systematic sample was visually interpreted using available high-resolution satellite images including a 5 m spatial resolution multispectral RapidEye mosaic^11^. As the spatial variability of forest structure and composition is high in mountains compared to the predominantly plain Terai region, two cluster designs were used (Fig. 3). Clusters in the mountainous physiographic regions consisted of 6 circular sample plots, whereas in the Terai, it was reduced to 4 circular plots.

In the second phase, a sub-sample of sample plots was selected from the first phase sample population using a two-phased stratified systematic cluster sampling design. Physiographic regions of Nepal were considered as strata. Inaccessible plots were excluded from the measurements, and a total of 2,038 sample plots were measured in the field. The inventory plots were distributed over 524 clusters. The plot centre was identified for each field plot using a handheld GPS (Garmin GPSMAP 64 s) and topographic maps (1:25,000 and 1:50,000 scale). A metal peg of 6-inch length was inserted at the plot centre and later covered with a thin layer of soil. The GPS unit was placed at the plot centre during measurements for way-point averaging, which provided a more accurate position of the plot centre using averages of several readings at the same location. Out of the total 2,038 sample plots, both forest AGB and SOC were estimated for 1,127 plots, only forest AGB for 882 plots and only forest SOC for 29 plots.

### Forest aboveground biomass (AGB)

#### Tree measurements

**Fig. 4:**
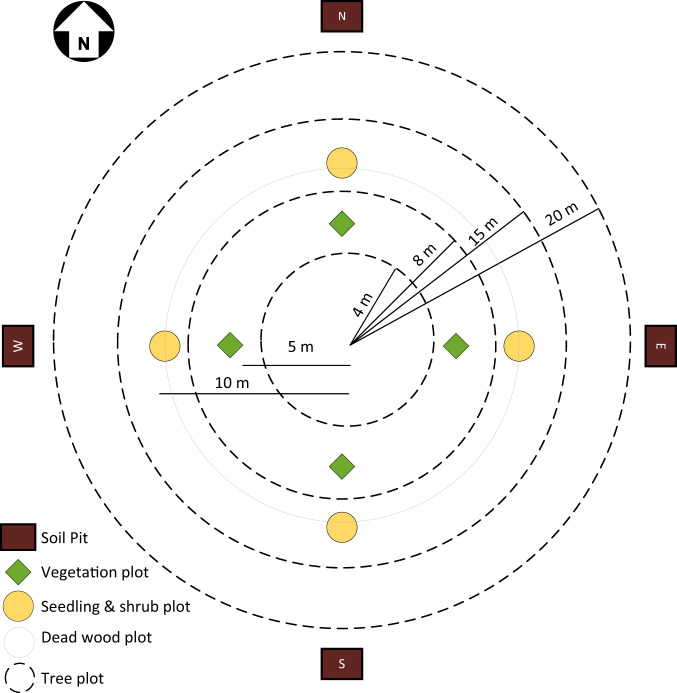
Layout of concentric sample plots for trees and sub-plots for soil and smaller vegetation.

Trees were measured in the concentric circular sample plots (CCSP) of different radii, while additional vegetation and soil samples were collected in four vegetation sub-plots, four shrubs and seedlings sub-plots, and four soil pits (Fig. 4). CCSP is the most commonly used sampling approach for national forest inventories across the globe^17^. In each circular plot of varying size, trees of specific diameter at breast height (DBH) classes were measured (Table 1). Sampling smaller areas for smaller trees reduces the cost and time associated with measuring a large number of smaller trees. The thresholds for the DBH limit and plot radius of the CCSP plot design (Table 1) were based on the analysis of forest structure from existing datasets.

**Table 1:** Details of sub-plots with different radii and areas within each concentric circular sample plot (CCSP) used to sample trees with varying thresholds of diameter at breast height (DBH).

Plot Radius (m)	DBH limit (cm)	Area (m2)
20	>30	1256.63
15	20.0–29.9	706.86
8	10.0–19.9	201.06
4	5.0–9.9	50.27

The previous version (2010) of the standard field measurement protocol^15^ provided guidelines to record details, including species codes, DBH, height, bearing and distance to sample trees from the plot centre. Tree height and distance from the plot centre were measured using a Haglöf range finder (Vertex IV and Transponder T3). DBH was measured at 1.3 m above the ground with 0.1 cm precision using a diameter tape. The bearing of sample trees from the plot centre was measured using a Suunto PM-5/360PC Clinometer.

#### Tree volume and biomass estimation

The volume of individual stems was estimated using field-measured DBH and the height of the tree. Stem volume over bark was calculated using an allometric equation^18^ (Eq. 1). For broken trees, the taper curve parameters^19^ and allometric equations^18^ were used to derive stem volume from stump height (15 cm) to the cut point of the tree.

1 $${\rm{ln}}({\rm{V}})={\rm{a}}+{\rm{b}}\cdot {\rm{ln}}\cdot {\rm{a}}({\rm{d}})+{\rm{c}}\cdot {\rm{ln}}\cdot {\rm{a}}({\rm{h}})$$

where, ln = Natural logarithm, V = Volume (m3), d = DBH (cm), h = Total tree height (m); a, b, and c are species-specific coefficients

Tree stem biomass was calculated using stem volume and density (Eq. 2) and species-specific wood density values^18,20^. The estimation of the tree branch and foliage biomass was based on published branch-to-stem, and foliage-to-stem biomass ratios^20^. The total biomass of an individual tree was estimated using Eq. 3. The plot-level forest AGB data presented in this paper included all standing trees (living, dead trees, broken trees and stumps) but excluded fallen deadwood and climbers.

2 $${{\rm{B}}}_{{\rm{s}}}={\rm{V}}\cdot {\rm{D}}$$

where, Bs = Stem biomass (kg), V = Stem volume (m3), and D = Air-dried wood density (kg m^−3^)

3 $${\rm{B}}={{\rm{B}}}_{{\rm{s}}}+{{\rm{B}}}_{{\rm{b}}}+{{\rm{B}}}_{{\rm{f}}}$$

where, B = Total tree biomass (kg), Bs = Stem biomass (kg), Bb = Branch biomass (kg), and Bf = Foliage biomass (kg)

### Forest soil organic carbon (SOC)

#### Forest soil sampling

**Fig. 5:**
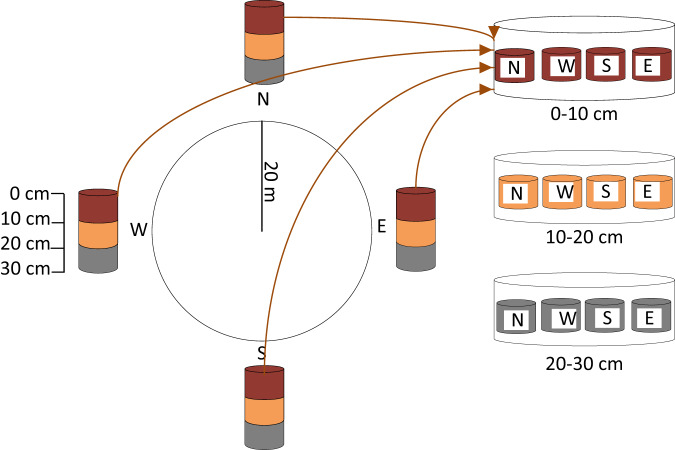
Soil sample collection from a sample plot. Each composite sample comprised soil samples for all cardinal directions at different depth classes.

The national forest inventory of Nepal (2010–2014) included soil sampling for the first time, with the objective of estimating national-scale forest SOC and filling the gap in understanding and reporting this carbon pool. To describe the soil profile, four soil pits were dug at 21 m from the CCSP plot centre in each cardinal direction, and soil samples were collected using a corer with dimensions of 100 mm long, a lower diameter of 37 mm (at cutting edge), and an upper diameter of 40 mm. Each soil sub-sample had a volume of 107.5 cm^3^. Composite soil samples were collected from three layers, namely 0–10 cm, 10–20 cm, and 20–30 cm depth, and stored in separate plastic bags for each layer (Fig. 5). The fresh mass of the composite sample was determined with an accuracy of 1 g. To estimate the relative volume of stones in the soil, the soil pit walls were observed based on the FAO Guidelines^21^.

In addition to soil sampling, various other attributes such as soil depth, organic layer type, organic layer thickness, and soil texture were recorded. Soil depth and classes were measured at random locations using a soil sampling auger with a 1 m shaft and classified according to the World Reference Base (WRB). To determine the mean penetration depth in cm, a 1 m long steel rod with five markings at 5 cm intervals was pushed to a depth of 30 cm. The mean depth was determined based on five pushes at the edge of CCSP. The type of organic layer (missing (0), raw humus (1), humus (2), Mull (3), and peat (4)) was recorded at each soil pit, and the thickness of the organic layer was measured with a reading accuracy of 1 cm at soil sampling pits.

#### Soil sample analysis

At the end of each forest measurement field mission, the samples were transported to the DFRS Soil Laboratory in Kathmandu, Nepal. Bulk density estimation was necessary to express SOC per unit volume and area, and it was determined by measuring the volume of the soil sample based on the corer, followed by air-drying and oven-drying to constant weight (105 ± 5° C for 24 hours). The total bulk density was immediately measured after oven-drying, and the sample was sieved through a 2 mm sieve to obtain the soil fine fraction (FF). The volume of the coarse fraction was determined using the water replacement method, and the bulk density of the soil fine fraction was calculated by subtracting the volume of the coarse fraction as stone particles are void of SOC. The SOC content was analysed using the partial wet combustion method^22^, with a correction factor of 1.33 to adjust for total organic carbon (OC) after the soil was passed through a 0.5 mm sieve for better homogenisation. The SOC in each 10 cm soil layer was calculated using the bulk density of the fine soil fraction for that layer.

To estimate the organic carbon content in the fine soil fraction (SOC_FF_), the dry soil bulk density (g cm^−3^) was multiplied by the proportion of SOC in the fine fraction of soil. However, this estimate included stones in the samples. To adjust for the stones, the proportion of stoniness (Stone %) determined during field sampling was used. The adjusted SOC_FF_ was calculated using Eq. 4. Once the SOC stock estimates were adjusted, they were extrapolated to a per-hectare amount and used to derive plot-level total SOC estimates for the 0–30 cm depth.

4 $${{\rm{SOC}}}_{{\rm{FF}}}^{{\rm{adj}}}=(100-{\rm{Stone \% }}/100)\cdot {{\rm{SOC}}}_{{\rm{FF}}}$$

where, ${{\rm{SOC}}}_{{\rm{FF}}}^{{\rm{adj}}}$ = Adjusted SOC_FF_, Stone% = Proportion of stoniness, and SOC_FF_ = Organic carbon content for fine soil fraction

## Data Records

The plot-level estimates of forest aboveground biomass (AGB) and soil organic carbon (SOC) stocks are available at 10.6084/m9.figshare.21959636.v1^23^. The estimates of these two major forest carbon pools, AGB and SOC are available for 2,009 and 1,156 inventory plots, respectively. The dataset includes a CSV (comma separated values) file with columns representing plot id, longitude, latitude, AGB (t ha^−1^) and SOC (t ha^−1^).

## Technical Validation

As the original datasets to derive the estimates of uncertainty were not available, a source of indicative errors could be the national forest resource assessment report^11^. The report has mentioned the use of standard error of the mean stem volume as an indicator of the reliability of tree measurements, whereby the target was set at a 95% confidence interval with a 10% margin of error for stem volume estimates. The standard error of the mean stem volume (m3 ha^−1^) was 6.17, equivalent to a 7.34% error of mean at a 95% confidence interval. In the case of forest SOC analysis, the quality assurance of the laboratory method was done using an inter-lab comparison of SOC analysis between the DFRS Soil lab in Nepal and the Metla Soil Lab in Finland. The DFRS lab used the Walkey-Black Wet Combustion method, while the Metla Soil Lab used dry combustion and LECO CHN (Carbon Hydrogen Nitrogen) Analyser^24^. Consistent results were reported between labs, particularly for organic carbon (OC%) ranging from 0 to 3% of OC). However, inconsistencies were observed for samples with larger OC% (>3%) as only one sample for this range was available, and thus DFRS lab approach was reported to be underestimated in the case of high SOC stocks^24^. The national allometry dataset for Nepal that we used provides volume equations and wood density for some major species and assumes average values for the rest, depending on the hills and plains. However, we anticipate that using species-specific allometric models and wood densities, if available, would help reduce uncertainties in our estimates. Despite the inclusion of the major forest carbon pools (AGB and SOC), the datasets included only a subset of total ecosystem carbon. There were missing records on litter for several plots in the raw data. Furthermore, the organic layer accounts for a relatively small proportion (0.67%) of the total SOC stock and contributes only 2% of the carbon content in the mineral soil^24^. Therefore, it was decided to exclude the organic layer component from the estimates of soil organic carbon (SOC). For fallen dead wood component though expected to have a rather small contribution to the total AGB, has not been included in this analysis. The input data for reporting on REDD+ and other initiatives would require including all stocks and fluxes. Therefore, the next step towards supporting a full understanding of the total carbon balance requires the inclusion and robust quantification of major stocks as well as fluxes.

## Usage Notes

The presented dataset of georeferenced plot-level estimates of forest AGB and SOC are calculated based on Nepal’s national forest inventory. Note that the plot-level estimates of forest AGB and SOC may vary from the official reporting by the FRTC due to differences in assumptions and calculation procedures. Researchers interested in the input data for the AGB and SOC estimates (i.e. tree-level and soil attributes data) need to inquire at the Forest Research and Training Center, Nepal.

## Data Availability

The calculation of plot-level forest AGB and SOC from the forest inventory data used R codes, which are accessible in Zenodo at 10.5281/zenodo.7809576^25^.

## References

[1] Miles L, Kapos V (2008). Reducing greenhouse gas emissions from deforestation and forest degradation: Global land-use implications. Science.

[2] Pistorius T (2012). From RED to REDD+: The evolution of a forest-based mitigation approach for developing countries. Current Opinion in Environmental Sustainability.

[3] Visseren-Hamakers IJ, Gupta A, Herold M, Peña-Claros M, Vijge MJ (2012). Will REDD+ work? The need for interdisciplinary research to address key challenges. Current Opinion in Environmental Sustainability.

[4] GFOI. Integration of remote-sensing and ground-based observations for estimation of emissions and removals of greenhouse gases in forests: Methods and Guidance from the Global Forest Observations Initiative. Tech. Rep., Food and Agriculture Organization, Rome. (2016).

[5] Hirata, Y., Takao, G., Sato, T. & Toriyama, J. REDD-plus cookbook: How to measure and monitor forest carbon. *REDD Research and Development Center, Forestry and Forest Products Research Institute, Tsukuba* (2012).

[6] Pelletier J, Martin D, Potvin C (2013). REDD+ emissions estimation and reporting: Dealing with uncertainty. Environmental Research Letters.

[7] Romijn E, Herold M, Kooistra L, Murdiyarso D, Verchot L (2012). Assessing capacities of non-Annex I countries for national forest monitoring in the context of REDD+. Environmental Science & Policy.

[8] Maraseni TN (2020). Mapping national REDD+ initiatives in the Asia-Pacific region. Journal of Environmental Management.

[9] Olson DM (2001). Terrestrial Ecoregions of the World: A New Map of Life on Earth: A new global map of terrestrial ecoregions provides an innovative tool for conserving biodiversity. BioScience.

[10] Stainton, J. D. A. *Forests of Nepal* (Hafner Publishing Company, 1972).

[11] DFRS. State of Nepal’s forests. Tech. Rep., Forest Resource Assessment (FRA) Nepal, Department of Forest Research and Survey (DFRS) (2015).

[12] Penman, J. *et al*. Good practice guidance for land use, land-use change and forestry. Tech. Rep., Institute for Global Environmental Strategies. (2003).

[13] Dixon RK (1994). Carbon pools and flux of global forest ecosystems. Science.

[14] Tyrrell, M. L., Ross, J. & Kelty, M. Carbon dynamics in the temperate forest. In Ashton, M. S., Tyrrell, M. L., Spalding, D. & Gentry, B. (eds.) *Managing Forest Carbon in a Changing Climate*, 77–107, 10.1007/978-94-007-2232-3_5 (Springer Netherlands, Dordrecht, 2012).

[15] FRTC. Field Manual, 2019 (remeasurement of permanent sample plot). Tech. Rep., Forest Resource Assessment (FRA), Forest Research & Training Center (FRTC), Nepal. 10.13140/RG.2.2.20785.45924 (2019).

[16] Kleinn, C. Forest resources inventories in Nepal: Status quo, needs, recommendations. Tech. Rep., His Majesty’s Government of Nepal, Kathmandu, Nepal & Finnish Forest and Park Service (1994).

[17] Lawrence, M., McRoberts, R. E., Tomppo, E., Gschwantner, T. & Gabler, K. Comparisons of national forest inventories. In Tomppo, E., Gschwantner, T., Lawrence, M. & McRoberts, R. E. (eds.) *National Forest Inventories: Pathways for Common Reporting*, 19–32, 10.1007/978-90-481-3233-1_2 (Springer Netherlands, Dordrecht, 2010).

[18] Sharma, E. R. & Pukkala, T. Volume and biomass prediction equations of forest trees of Nepal, 10.5281/zenodo.7810164 (1990).

[19] Heinonen J, Saramki J, Sekeli PM (1996). A polynomial taper curve function for Zambian exotic tree plantations. Journal of Tropical Forest Science.

[20] MPFS. Master plan for the forestry sector (MPFS). Main report. Government Document, Ministry of Forests and Soil Conservation, Nepal (1989).

[21] FAO. Guidelines for soil description. Tech. Rep. 9251055211, Food and Agriculture Organization of the United Nations, Italy (2006).

[22] Walkley A, Black IA (1934). An examination of the Degtjareff method for determining soil organic matter, and a proposed modification of the chromic acid titration method. Soil science.

[23] Khanal S, Boer MM (2023). figshare.

[24] DFRS. Terai forest of Nepal 2010 to 2012. Tech. Rep., Forest Resource Assessment (FRA) Nepal, Department of Forest Research and Survey (DFRS) (2014).

[25] Khanal S (2023). Zenodo.

